# Emerging roles for lncRNA-NEAT1 in colorectal cancer

**DOI:** 10.1186/s12935-022-02627-6

**Published:** 2022-06-08

**Authors:** Shirin Azizidoost, Farhoodeh Ghaedrahmati, Omid Anbiyaee, Riyadh Ahmad Ali, Maryam Cheraghzadeh, Maryam Farzaneh

**Affiliations:** 1 Atherosclerosis Research Center, Ahvaz Jundishapur University of Medical Sciences, Ahvaz, Iran; 2 Department of Immunology, School of Medicine, Isfahan University of Medical Sciences, Isfahan, Iran; 3 Cardiovascular Research Center, Nemazi Hospital, School of Medicine, Shiraz University of Medical Sciences, Shiraz, Iran; 4 Department of Medical Laboratory Science, College of Health Science, Lebanese French University, Kurdistan Region, Iraq; 5 Department of Biochemistry, School of Medicine, Ahvaz Jundishapur University of Medical Sciences, Ahvaz, Iran; 6 Fertility, Infertility and Perinatology Research Center, Ahvaz Jundishapur University of Medical Sciences, Ahvaz, Iran

**Keywords:** Colorectal cancer, lncRNAs, NEAT1, Progression

## Abstract

Colorectal cancer (CRC) is the third cause of cancer death in the world that arises from the glandular and epithelial cells of the large intestine, during a series of genetic or epigenetic alternations. Recently, long non-coding RNAs (lncRNAs) has opened a separate window of research in molecular and translational medicine. Emerging evidence has supported that lncRNAs can regulate cell cycle of CRC cells. LncRNA NEAT1 has been verified to participate in colon cancer development and progression. NEAT1 as a competing endogenous RNA could suppress the expression of miRNAs, and then regulate molecules downstream of these miRNAs. In this review, we summarized emerging roles of NEAT1 in CRC cells.

## Introduction

Colorectal cancer (CRC) is one of the most prevalent cancers and the third leading cause of cancer death in both men and women [1, 2]. CRC starts at the inner lining of the colon, rectum, and appendix [3, 4]. In 2020, there will be over 10,000 new cases, with a growing number of young individuals [5]. A variety of etiological factors may have a role in the development of CRC, including environmental, genetic, and epigenetic factors [1, 6]. Based on the genetics and etiology of the disease, CRC is commonly classified into sporadic, hereditary, or familial [7, 8]. Sporadic disease, in which there is no family history, accounts for about 70% of all CRC [9]. The majority of CRCs are patients over 50 years old, dietary, environmental factors, and genetic changes in the adenoma-carcinoma sequences [10–12]. There are fewer than 10% of patients who have an inherited predisposition to CRC, and these cases are subdivided based on the presence of polyps (level 0 to 4) [13]. These conditions have high risks of developing CRC, and many of them have underlying genetic mutations [14]. Familial CRC is the third and least well understood pattern [15]. A family history of CRC occurs in up to 25% of affected patients but the pattern is not consistent with those of the inherited syndromes [16]. Individuals from these families are more likely to develop CRC, although the risk is not as high as those with inherited syndromes [17]. Despite substantial advances in current CRC treatments such as adjuvant chemo-radiotherapies and immunological therapy, the prognosis for patients with the advanced-stage disease remains poor and the 5-year survival rate remains unsatisfactory [18, 19]. To increase the survival rate of affected individuals, a better knowledge of the processes of CRC beginning and development is urgently required and more research is needed to identify and develop new biomarkers and targets for its diagnosis and treatment [20–22]. Recently, it has been shown that long non-coding RNAs (lncRNAs) are participated in the pathogenesis of CRC [23]. LncRNAs are non-coding RNA molecules with a length of more than 200 nucleotides without protein-coding potential [24]. Although they lack coding ability, the majorities of them are transcribed by RNA polymerase II and share similarities with messenger RNAs (mRNAs) [25]. LncRNAs as an initially transcribed RNA or a mature spliced RNA can regulate the expression of significant genes at multiple levels through epigenetic regulation and by modulating transcription, post-transcriptional processes, translation, and protein modification [26]. Additionally, lncRNAs by targeting microRNAs (miRNAs) play critical roles in physiological processes such as development, tissue differentiation, reproduction, immunity, tumor formation, and development [27, 28]. miRNAs are a small single-stranded non-coding RNAs that play fundamental roles in gene expression and posttranscriptional gene silencing [29, 30]. LncRNAs also play a critical role in peripheral blood components, including serum and plasma [31]. Some of these lncRNAs are upregulated in tumors and act as oncogenes, while others serve as tumor suppressors [32]. There is an association between overexpressed lncRNAs and poor prognoses and metastasis in CRC patients [33]. These findings support the idea that lncRNAs are important therapeutic targets in CRC (Table 1). Furthermore, lncRNA-mediated treatment of patients with CRC might be a promising approach [34]. LncRNAs play a key role in colon carcinogenesis and progression [35, 36], with one study used RNA sequencing data from the TCGA dataset to find approximately 200 differently expressed lncRNAs in CRC [37]. The patient outcome, cell proliferation, apoptosis, metastasis, invasion, cell cycle, epithelial-mesenchymal transition (EMT), cancer stem cells (CSCs), and drug resistance are controlled by lncRNAs [38]. Nuclear enriched abundant transcript 1 (NEAT1) is a novel lncRNA that participated in a variety of cancers, such as breast, gastric, and lung [39–41]. Accumulating evidences reported that NEAT1 play critical roles in the tumorigenesis of CRC [42]. In the present manuscript we summarized functional roles of NEAT1 in the pathogenesis and progression of CRC.

**Table 1:** Functional roles of the NEAT1/miRNA/transcription factor axes in colorectal cancer

NEAT1/miRNA/transcription factor	Cell line (in vitro)	Animal model (in vivo)	Patients-derived tissue	Si-lncRNA- targeted therapy	Cancer cell initiation (proliferation)	Cancer cell progression	Cancer cell metastasis	Cancer cell apoptosis	Refs.
MiR-34a/SIRT1/Wnt/β-catenin	✓	–	✓	✓	–	✓	✓	–	[60]
MiR-185-5p/IGF2	✓	–	✓	✓	–	✓	–	–	[65]
MiR-495-3p/CDK6	✓	✓	–	✓	✓	✓	–	✓	[66]
MiR-193a-3p	✓	✓	✓	✓	–	✓	–	–	[68]
MiR-205-5p/VEGFA	✓	–	✓	✓	✓	✓	–	–	[69]
MiR-196a-5p/GDNF	✓	–	–	✓	✓	–	–	–	[71]
DDX5/Wnt/β-catenin	✓	✓	✓	✓	✓	✓	✓	–	[74]
MiR-150-5p/CPSF4	✓	–	✓	✓	–	✓	–	✓	[76]
MiR-138/SLC38A1	✓	✓	✓	✓	✓	✓	–	✓	[78]
KDM5A/Cul4A/Wnt/β-catenin	✓	✓	✓	✓	–	✓	–	–	[79]
MiR-486 5p/NR4A1/Wnt/β-catenin	✓	–	✓	✓	✓	✓	–	✓	[85]
MiR-193a-3p/KRAS	✓	–	✓	✓	✓	–	–	✓	[86]
Akt	✓	–	✓	✓	✓	–	–	✓	[58]
ALKBH5	✓	✓	✓	✓	✓	✓	✓	✓	[93]
MiR-377-3p	✓	–	✓	✓	✓	–	–	✓	[99]

## Characteristics of NEAT1

**Fig. 1:**
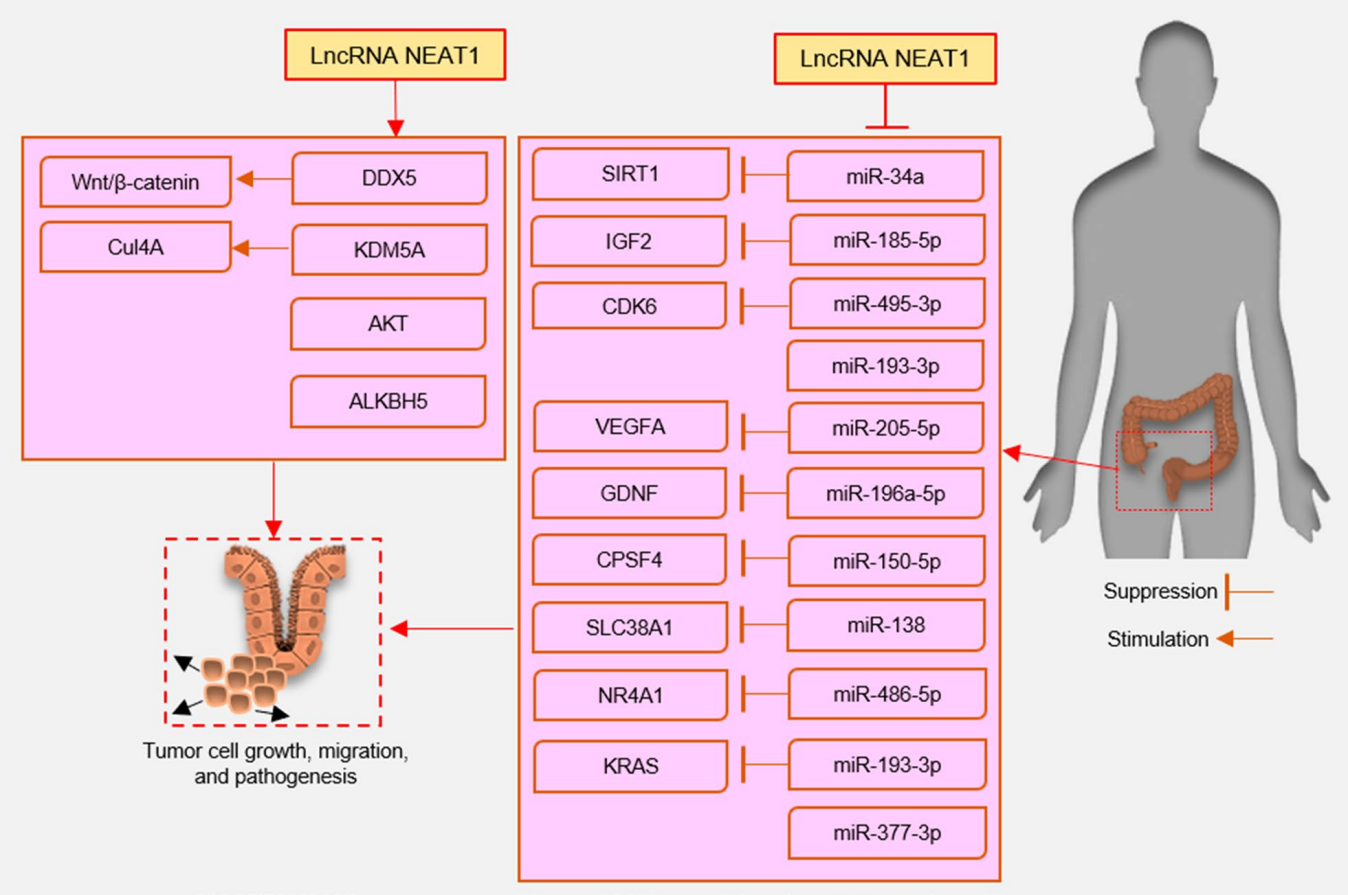
Functional roles of NEAT1 in colorectal cancer. LncRNA NEAT1 by targeting several miRNA/transcription factors axes induced colorectal cell tumorigenesis

Nuclear paraspeckle assembly transcript 1 (NEAT1) is a lncRNA that is transcribed by RNA polymerase II from the familial tumor syndrome multiple endocrine neoplasia (MEN) type 1 loci on chromosome 11q13.1, encodes two transcriptionally distinct variants, NEAT1-1 (3756 bp) and NEAT1-2 (22,743 bp) [43]. Two isoforms share an identical promoter and 5′-end but they differ at the 3′-end, thus making them different subtypes. This lncRNA assists in the formation and assembly of nuclear paraspeckles, a group of highly dynamic nuclear subdomains [44, 45]. Paraspeckles have a core–shell spheroidal structure and the middle part of NEAT1-2 forms a core, which is encircled by NEAT1-1 and the NEAT1-2, -5′ and 3′-ends [46]. In the formation of paraspeckles, NEAT1-2 has been implicated strongly [47, 48]. Paraspeckle protein (PSP) 1, PSP2 and p54^nrb^ bind to the NEAT1 transcriptional start site to create paraspeckles [47, 49]. These nuclear bodies could act as a “reservoir” for mRNA retention in the nucleus. They’ve also been demonstrated to migrate to the cytoplasm and modulate the function of cytoplasmic proteins and RNA [47]. While NEAT1 is mostly identified in the nucleus, a small amount is also found in the cytoplasm, and this contains miRNA-binding sites that allow it to regulate and communicate with mRNAs by competing for shared miRNAs [39, 41, 50, 51]. miRNAs are a group of small noncoding regulatory RNA molecules that have modulatory roles in a variety of biological processes [52]. Dysregulation of miRNA would have a significant impact on cancer development [53]. Evidence has shown that NEAT1 not only participated in vital physiological processes, including immune responses, organogenesis, and myogenesis, but also plays an important role in pathological processes [54]. The NEAT1 gene exhibits characteristics of cancer drivers since it initiates and progresses tumors, and its frequent dysregulation in cancer is correlated with metastasis, recurrence rate, and survival [55]. There has recently been a great deal of interest in lncRNAs for potential functions in the different stages of CRC formation, invasion, and progression [56]. According to recent studies, NEAT1 is overexpressed and plays an oncogenic role in CRC [57–59]. Here, we described how this lncRNA contributes to the biological process of CRC (Fig. 1).

## Functional roles of NEAT1 in colorectal cancer

### NEAT1/miR-34a/SIRT1/Wnt/β-catenin

It has been reported that NEAT1 expression was dramatically induced in tissues of patients with CRC in comparison to those with normal tissues. High expression of NEAT1 is correlated with clinicopathological significance of CRC including the tumor size, distant metastasis, decreased overall survival, and disease-free survival rate. Moreover, NEAT1 induced the proliferative and invasion activity of CRC cells. So, upregulated NEAT1 may be a predictive risk factor for CRC diagnosis and prognosis [60]. It has been shown that NEAT1 via direct targeting of miR-34a can induce CRC [60, 61]. miR-34a has the tumor suppression effect on silent information regulator 1 (SIRT1) in CRC [62]. SIRT1 as a member of the Sirtuin family not only regulates different cellular biological events but also upregulates in a variety of cancers including CRC [63, 64]. Recent findings indicated that SIRT1 expression has a positive and negative correlation with NEAT1 and miR-34a, respectively. So, NEAT1 increased SIRT1 expression through targeting miR-34a, thereby inducing pathogenesis of CRC. Furthermore, crucial function of the SIRT1/Wnt/β-catenin signaling pathway-dependent manner has been reported in different cell process. Overexpression of NEAT1 increased SIRT1 and Wnt/β-catenin member expressions, whereas miR-34a mimics reversed the NEAT1-induced overexpression of SIRT1 and Wnt/β-catenin. Altogether, NEAT1 promoted pathogenesis of CRC via the miR-34a/SIRT1/Wnt/β-catenin axis, thereby standing as a promising biomarker for the management of CRC [60].

### NEAT1/miR-185-5p/IGF2

A study reported that high expression of NEAT1 was associated with lower overall survival of patients with CRC. Such upregulation increased invasion along with migration of colorectal malignant cells through overexpression of vimentin and downregulation of cytokeratin 19 and E-cadherin as EMT-correlated genes. Based on RNA pull-down analysis, there was a special interaction between NEAT1 and miR-185-5p. Insulin-like growth factor 2 (IGF2) was found to function as the main target of NEAT1 and miR-185-5p. NEAT1 by targeting IGF2 stimulated the pathogenesis of CRC [65].

### NEAT1/miR-495-3p/CDK6

In contrast to overexpression of NEAT1 in CRC, miR-495-3p as a predicted target of NEAT1 was downregulated. Knockdown of NEAT1 using LV-shNEAT1 repressed CRC cell proliferation along with inhibited expression of cyclinD1 and cyclin E as cell cycle-correlated protein markers. Therefore, NEAT1 suppression promoted apoptosis and inhibited migration and invasion of CRC cells. Such biological function of inhibited NEAT1 is reported to be regulated through promoting miR-495-3p [66]. Cyclin-dependent kinase 6 (CDK6) which is modulated by cyclins was identified as a possible target of miR-495-3p and its expression was regulated in a negative manner by miR-495-3p. As a result, NEAT1 participated in CRC development via sponging miR-495-3p and promoting CDK6 expression [66, 67].

### NEAT1/miR-193a-3p

High expression of NEAT1 was demonstrated to be associated with clinicopathological significance of CRC, including poor prognosis, TNM stage, low survival, and high cancer recurrence. Silencing NEAT1 was revealed to induce E-cadherin protein expression and reduce N-cadherin and Vimentin levels, thereby decreasing the invasion of CRC cells. Further analysis showed other biological functions of NEAT1 knockdown such as inhibited proliferation, colony formation potential, and increased apoptosis of CRC. Furthermore, inhibition of miR-193a-3p as a sponge target of NEAT1 has been suggested to induce proliferation and invasion of CRC cells. Hence, the oncogenic function of NEAT1 induced CRC tumorigenesis through targeting miR-193a-3p [68].

### NEAT1/miR-205-5p/VEGFA

High expression of both NEAT1 and vascular endothelial growth factor A (VEGFA), and low expression of miR-205-5p have been proved in CRC [69]. VEGFA as key regulator of angiogenesis [70], cancer progression, and metastasis has been found to be a direct target of miR-205-5p. Upregulation of NEAT1 intensified cancer growth by regulating miR-205-5p. Besides, downregulation of NEAT1 and VEGFA not only suppressed the proliferative activity of CRC cells but also decreased matrix metalloproteinase-2 (MMP2) and MMP9 as cell-migration- and invasion-correlated proteins. So, upregulated NEAT1 induced CRC pathogenesis by modulating the miR-205-5p/VEGFA pathway, thereby suggesting being an intriguing marker in CRC therapy and diagnosis [69].

### NEAT1/miR-196a-5p/GDNF

An increase of cell proliferation and migration in CRC cells was reported following NEAT1 overexpression. Direct regulation of miR-196a-5p by NEAT1 and their inverse expressions in CRC revealed that miR-196a-5p along with NEAT1 participated in CRC pathogenesis [71]. Further investigation demonstrated that miR-196a-5p represented its function through modulation of glial cell line-derived neurotrophic factor (GDNF) as a neurotrophic factor affecting tumor invasion and metastasis [71, 72]. It can be concluded that NEAT1 exerted its regulatory mechanism in CRC pathogenesis through miR-196a-5p inhibition and GDNF induction.

### NEAT1/DDX5/Wnt/β-catenin

Downregulated NEAT1 has been reported to reduce the proliferative activity of CRC cells, increased poly (ADP-ribose) polymerase-1 (PARP-1) and cleaved caspase-3 as hallmark of apoptotic proteins [71, 73, 74]. Furthermore, NEAT1 repression led to downregulate MMP2, MMP9, and N-cadherin along with E-cadherin upregulation. Hence, NEAT1 increased proliferation and metastasis of CRC cells by attenuating apoptosis [74]. Since the discovery of direct binding of DEAD box helicase 5 (DDX5) as a key protein involved in tumorigenesis [75], recent finding revealed NEAT1 modulated DDX5 stability in a direct binding manner, consequently activated Axin2, c-myc, and cyclinD1 as the Wnt/β-catenin pathway targets. It is suggested that NEAT1 elevated CRC pathogenesis through activating the Wnt/β-catenin pathway in a DDX5-regulated manner. Recent pharmacological approaches could focus on the NEAT1/DDX5/Wnt/β-catenin axis as a possible therapeutic axis in CRC [74].

### NEAT1/miR-150-5p/CPSF4

The inverse expression trend between NEAT1 and miR-150-5p in CRC could predict their possible correlation. NEAT1 silencing promoted the 5-fluorouracil (5-Fu) sensitivity and apoptosis and repressed the invasion and the expression of resistance-correlated proteins including P-gp and GST-π in CRC cells. It was found that inhibition of miR-150-5p as a target of NEAT1 reversed NEAT1 silencing on the progression of CRC [76]. Moreover, upregulation of cleavage and polyadenylation specific factor 4 (CPSF4) as a target of miR-150-5p reversed the NEAT1 silencing effect on CRC pathogenesis [76, 77]. Further investigation indicated that NEAT1 promoted CPSF4 expression through targeting miR-150-5p to elevate CRC progression. The NEAT1/miR-150-5p/CPSF4 network highlighted new approach for CRC drug resistance [76].

### NEAT1/miR-138/SLC38A1

The expression of solute carrier family 38 member 1 (SLC38A1) as a tumor-inducing agent has been found to be upregulated in CRC. NEAT1 silencing or SLC38A1 low expression prevented the proliferative and invasion ability of CRC cells but induced CRC apoptosis and autophagy. In addition, NEAT1 modulated SLC38A1 expression through sponging miR-138. Therefore, knockdown of NEAT1 suppressed CRC cells tumor growth and progression with the miR-138/SLC38A1 axis, exerting an underlying strategy for CRC management [78].

### NEAT1/KDM5A/Cul4A/Wnt/β-catenin

NEAT1 silencing repressed the malignant features of CRC cells [79]. Lysine-specific demethylase 5A (KDM5A) which is participated in human cancer has been inhibited through binding of NEAT1 to the E2F transcription factor 1 (E2F1) protein [79, 80]. E2F1 is known to be associated with the pathogenesis, metastasis, and chemoresistance of CRC cells [81]. KDM5A also suppressed cullin 4A (Cul4A) as a ubiquitin ligase promoting tumorigenesis [82]. Cul4A upregulation facilitated the malignant features of the si-NEAT1-transfected CRC cells. Besides, activation of the Wnt pathway through KDM5A/Cul4A is exerted via NEAT1 [79]. Regarding dysregulated the Wnt pathway in CRC development [83], NEAT1 promoted CRC progression through the KDM5A/Cul4A/Wnt axis [79].

### NEAT1/miR-486-5p/NR4A1/Wnt/β-catenin

Nuclear orphan receptor 4A1 (NR4A1) as a pro-oncogenic and poor prognosis factor for CRC survival has been overexpressed in CRC [84, 85]. NEAT1 or NR4A1 loss of function suppressed the proliferation along with motility but promoted apoptosis of CRC cells. In contrast, NR4A1 knockdown-mediated impacts on CRC cells. Considering miR-486-5p targeting by NEAT1 and the role of NEAT1 in inducing β-catenin, c-myc, and cyclinD1, this lncRNA facilitated the progression of CRC through sponging miR-486-5p to regulate the NR4A1/Wnt/β-catenin pathway [85].

### NEAT1/miR-193a-3p/KRAS

High expression of NEAT1 along with low expression of miR-193a-3p as a target of NEAT1 has been reported in CRC. Downregulated NEAT1 impaired the viability of CRC cells whereas miR-193a-3p inhibition increased tumor cell proliferation and migration, and reduced CRC apoptosis by targeting Kirsten rat sarcoma viral oncogene homology (KRAS) expression [86]. KRAS as a major pathway in tumorigenesis was introduced as a downstream target of miR-193a-3p [86, 87]. NEAT1 silencing or miR-193a-3p induction prevented CRC progression by controlling KRAS expression. The NEAT1/miR-193a-3p/KRAS network could exert intriguing advancement for diagnostic and management of CRC [86].

### NEAT1/Akt

A recent study demonstrated that NEAT1 silencing resulted in low expression of Bcl2 and high expression of Bax in CRC cells, which are involved in cell growth and apoptosis [58]. Based on the roles of Akt signaling in cell growth and apoptosis [88], recent finding revealed that NEAT1 knockdown prevented Akt phosphorylation at Ser473, thereby repressing Akt activation [58]. Considering Akt activation in CRC progression [89], NEAT1 affected the proliferation and apoptosis of CRC via modulation of the Akt pathway and the NEAT1/Akt pathway may act as a possible target for cancer therapy [58].

### NEAT1/ALKBH5

RNA modification like N6-methyladenosine (m6A) is regarded as the most leading way of gene expression regulation and unusual changes of such modification led to cancer recurrence [90–92]. Silencing ALKBH5 as a demethylated enzyme of m6A was reported to reduce the tumor behavior of CRC cells and inhibited the expression of proliferating cell nuclear antigen (PCNA) and the migration induced by NEAT1. Knockdown of ALKBH5 promoted cell apoptosis of CRC cells. Thus, the NEAT1/ALKBH5 axis may regard as possible therapeutic target for CRC management [93].

### NEAT1/miR-377-3p

Small interfering RNA (siRNA)-related therapies are a possible approach for targeting different cancers but loss of a desired delivery system still restricts their development [94–96]. Chitosan nanoparticles (CNPs) have been developed for targeted delivery of siRNA [97, 98]. In a recent finding, nano-NEAT1 siRNA was shown to reverse upregulated NEAT1 in CRC along with decreased CRC viability. Moreover, nano-NEAT1 siRNA therapy significantly induced CRC apoptosis through upregulating Bax as pro-apoptotic protein and downregulating Bcl-2 as an anti-apoptotic marker [99, 100]. Furthermore, inhibition of miR-377-3p as a target of NEAT1 could neutralize NEAT1 knockdown properties in CRC. Hence, the NEAT1/miR-377-3p axis might be a promising candidate for CNP-based therapy for siRNA-related CRC gene management [99].

## Clinical significance of NEAT1

Chemotherapy has been demonstrated to diminish cancer recurrence and life quality of CRC patients but chemotherapy resistance is known as a poor prognosis and recurrence factor in CRC [101]. 5-FU stands as an efficient drug for CRC and knowledge of its resistance in recovering CRC survival is unknown [102, 103]. Silencing autophagy as a regulator of lysosomal degradation phenomenon has been reported to promote the efficacy of chemotherapy [104, 105]. Recent findings revealed that NEAT1 targeted miR-34a to induce autophagy, thereby facilitating chemoresistance of 5-FU in CRC. Knockdown of NEAT1 and consequent inhibited autophagy can be a chemotherapeutic approach to increase CRC sensitivity of 5-FU through targeting miR-34a [106]. Besides, CSCs are introduced as an important cause of chemoresistance [107, 108]. NEAT1 silencing was found to repress the ALDH1 activity and CD133 expression in CRC cells, thereby affecting CRC stemness maintenance. NEAT1 is also involved in chromatin remodeling, histone acetylation, and the expression of ALDH1 and c-Myc as the stemness-related genes. Co-expression of NEAT1/ALDH1/c-Myc is associated with 5-FU resistance, recurrence, and poorer prognosis of CRC patients [101]. Altogether, NEAT1 may be regarded as a promising approach for the therapy of CRC drug resistance. According to previous finding, the miR-124/iASPP/p53 axis showed significant impact on CRC photodynamic therapy (PDT) resistance [109, 110]. NEAT1 knockdown facilitated the sensitivity of CRC cells to PDT and suppressed the effect of PDT on CRC growth through sponging miR-124It has been demonstrated that the p53 deletion or mutation can induce CRC cells resistance to PDT. The c-Myc/miR-124/NEAT1/p53/iASPP axis exerted their regulatory role in CRC response to the chemotherapy [111]. It was revealed that cleavage of gasdermin E (GSDME) as a member of the gasdermin family altered apoptosis to pyroptosis as a lytic cell death which is correlated with chemotherapy along with anticancer immunity in CRC [112–115].

Recent findings showed that ionizing radiation (IR) of CRC induced caspases and Bax-regulated apoptosis along with GSDME-regulated pyroptosis. The luciferase reporter assay revealed GSDME was a downstream target of miR-448. Hence, high expression of NEAT1 reduced the expression of miR-448 and increased GSDME expression levels. Altogether, NEAT1 induced radioresistance of CRC cells through promoting IR-increased pyroptosis by modulating GSDME expression. The NEAT1/miR-448/GSDME axisparticipated in radioresistance of CRC [117]. Therefore, NEAT1 is implicated in the pathogenesis of CRC through regulating the chemo and radioresistance of CRC cells. Clinical significance of NEAT1 expression displayed its positive association with tumor size, TNM stage, carcinoembryonic antigen (CEA) level, lymphatic metastasis, and presence of distant metastasis of CRC [60]. Among clinicopathologic features of CRC, CEA level along with TNM stage have been reported to be dramatically correlated with overall survival of patients with CRC [118]. Moreover, high expression of NEAT1 was associated with poorer disease-free survival as well as tumor recurrence in tumor tissues of CRC patients, thereby highlighting NEAT1 as a possible predictive marker for the diagnosis and prognosis of CRC patients [101].

## Conclusion

NEAT1 plays a role in chemo-resistance, tumor growth, and metastasis, according to several lines of evidence. This lncRNA induced stem cell properties in tumoral tissues, as well. NEAT1 could regulate a variety of miRNAs and their target genes to stimulate tumorigenesis. In contrast to solid tumors, NEAT1 expression is downregulated in acute promyelocytic leukemia and functions as a tumor suppressor by promoting leukocyte differentiation. In solid tumors and haematological malignancies, different gene expression patterns may be responsible for this discrepancy. Such different roles should be considered in designing specific treatments for each type of cancer. This lncRNA is considered as a potential biomarker in several cancer types and developing NEAT1-targeting therapies might be a novel strategy against CRC. CRISPR/Cas-9 genome editing technology may be used to target NEAT1 loci for therapeutic purposes, but there are still challenges, such as the risk of disease occurrence due to unwanted mutations, the immune response to the delivery system, and other toxic side effects. In addition, evaluation of its expression levels in cancer patients' serum in order to replace invasive biopsy with liquid biopsy should be evaluated. Although the chemical stability of NEAT1 in biological samples (e.g., serum) is also unknown. Further studies are required to determine the exact mechanism of NEAT1 involved in carcinogenesis and the underlying mechanism of NEAT1 dysregulation in human cancers.

## Data Availability

The datasets used and/or analyzed during the current study are available from the corresponding author on reasonable request.

## Abbreviations

CNPs: Chitosan nanoparticles
CSCs: Cancer stem cells
CRC: Colorectal cancer
CPSF4: Cleavage and polyadenylation specific factor 4
Cul4A: Cullin 4A
DDX5: DEAD box helicase 5
EMT: Epithelial-mesenchymal transition
E2F1: E2F transcription factor 1
GDNF: Glial cell line-derived neurotrophic factor
GSDME: Cleavage f gasdermin E
IR: Ionizing radiation
incRNAs: Intergenic lncRNAs
IGF2: Insulin-like growth factor 2
lncRNAs: Long non-coding RNAs
KDM5A: Lysine-specific demethylase 5A
KRAS: Kirsten rat sarcoma viral oncogene homology
mRNAs: Messenger RNAs
MMP2: Matrix metalloproteinase-2
m6A: N6-methyladenosine
NR4A1: Nuclear orphan receptor 4A1
PARP-1: Poly (ADP-ribose) polymerase-1
PDT: Photodynamic therapy
PCNA: Proliferating cell nuclear antigen
SLC38A1: Solute carrier family 38 member 1
siRNA: Small interfering RNA
SIRT1: Silent information regulator 1
TILs: Tumor infiltrating lymphocytes
VEGFA: Vascular endothelial growth factor A
5-FU: 5-Fluorouracil
